# Low in-hospital mortality rate in patients with COVID-19 receiving thromboprophylaxis: data from the multicentre observational START-COVID Register

**DOI:** 10.1007/s11739-021-02891-w

**Published:** 2022-01-01

**Authors:** Daniela Poli, Emilia Antonucci, Walter Ageno, Paolo Prandoni, Gualtiero Palareti, Rossella Marcucci, the START-COVID Investigators, Rossella Marcucci, Daniela Poli, Walter Ageno, Giovanna Colombo, Chiara Ambaglio, Guido Arpaia, Giovanni Barillari, Giuseppina Bitti, Eugenio Bucherini, Antonio Chistolini, Alessandra Serrao, Egidio De Gaudenzi, Valeria De Micheli, Anna Falanga, Teresa Lerede, Luca Barcella, Laura Russo, Silvia Galliazzo, Alberto Gandolfo, Gianni Biolo, Valentina Trapletti, Giorgio Ghigliotti, Elisa Grifoni, Luca Masotti, Egidio Imbalzano, Gianfranco Lessiani, Niccolò Marchionni, Giuliana Martini, Sara Merelli, Franco Mastroianni, Giovanni Larizza, Carlo Nozzoli, Serena Panarello, Chiara Fioravanti, Simona Pedrini, Federica Bertola, Raffaele Pesavento, Filippo Pieralli, Pasquale Pignatelli, Daniele Pastori, Paola Preti, Elias Romano, Alessandro Morettini, Girolamo Sala, Fabrizio Foieni, Michela Provisone, Luca Sarti, Antonella Caronna, Federico Simonetti, Ilaria Bertaggia, Piera Sivera, Carmen Fava, Viviana Scancassani, Michele Spinicci, Alessio Bartoloni, Adriana Visonà, Beniamino Zalunardo, Sabina Villalta

**Affiliations:** 1 Fondazione Arianna Anticoagulazione, Bologna, Italy; 2 Dipartimento di Medicina e Chirurgia, Università dell’Insubria, Varese, Italy; 3 Department of Experimental and Clinical Medicine, University of Florence, Firenze, Italy; 4 Present Address: Centro Trombosi, Azienda Ospedaliero Universitaria Careggi, Viale Morgagni, 85–50134 Firenze, Italy

**Keywords:** COVID-19 disease, Antithrombotic, Heparin, Mortality

## Abstract

COVID-19 infection causes respiratory pathology with severe interstitial pneumonia and extra-pulmonary complications; in particular, it may predispose to thromboembolic disease. The current guidelines recommend the use of thromboprophylaxis in patients with COVID-19, however, the optimal heparin dosage treatment is not well-established. We conducted a multicentre, Italian, retrospective, observational study on COVID-19 patients admitted to ordinary wards, to describe clinical characteristic of patients at admission, bleeding and thrombotic events occurring during hospital stay. The strategies used for thromboprophylaxis and its role on patient outcome were, also, described. 1091 patients hospitalized were included in the START-COVID-19 Register. During hospital stay, 769 (70.7%) patients were treated with antithrombotic drugs: low molecular weight heparin (the great majority enoxaparin), fondaparinux, or unfractioned heparin. These patients were more frequently affected by comorbidities, such as hypertension, atrial fibrillation, previous thromboembolism, neurological disease, and cancer with respect to patients who did not receive thromboprophylaxis. During hospital stay, 1.2% patients had a major bleeding event. All patients were treated with antithrombotic drugs; 5.4%, had venous thromboembolism [30.5% deep vein thrombosis (DVT), 66.1% pulmonary embolism (PE), and 3.4% patients had DVT + PE]. In our cohort the mortality rate was 18.3%. Heparin use was independently associated with survival in patients aged ≥ 59 years at multivariable analysis. We confirmed the high mortality rate of COVID-19 in hospitalized patients in ordinary wards. Treatment with antithrombotic drugs is significantly associated with a reduction of mortality rates especially in patients older than 59 years.

## Introduction

The coronavirus disease of 2019 (COVID-19) is a viral illness caused by the RNA betacoronavirus severe acute respiratory syndrome coronavirus 2 (SARS-CoV2). The first pneumonia cases of unknown origin were identified in Wuhan, China, in December 2019. After few weeks, the World Health Organization (WHO) declared the pandemic. (WHO Director-General's opening remarks at the media briefing on COVID-19–11 March 2020).

After the diffusion in China, Italy was the first country severely interested by the pandemic, with a widespread diffusion especially in Northern Italy [1]. COVID-19 infection causes respiratory pathology with severe interstitial pneumonia, but also causes several extra-pulmonary complications. In particular, COVID-19 may predispose to both venous and arterial thromboembolic disease due to excessive inflammation, hypoxia, and immobilization. Moreover, it has been described a COVID-19-associated coagulopathy that may further increase the thrombotic risk [2–5]. COVID-19 patients may show marked increase of inflammatory markers, as well as signs of endothelial dysfunction, platelet activation, and hypercoagulability [6]. The mortality rate is high, even if differences have been reported across published studies, ranging from 16 to 78% of patients who required hospital admission. Several factors associated with a high risk for death in these patients were described, in particular comorbidities such as hypertension, coronary heart disease and diabetes [7–11]. Among, predictors of mortality, also COVID-19 associated coagulopathy has been associated with poor outcome [2, 12, 13]. Given the high risk of venous thromboembolism and the role of coagulopathy on patient outcomes, current guidelines recommend the use of thromboprophylaxis for patients with COVID-19 [14–17]. However, the optimal dosage of heparin treatment is not known and the need for intermediate or therapeutic heparin doses has been proposed. However, there is concern on the bleeding risk associated with the higher heparin dosage [16, 18–20].

We conducted a multicentre, Italian nationwide, retrospective, observational study on COVID-19 patients admitted to ordinary wards, to describe the demographics, baseline comorbidities, laboratory tests at presentation, bleeding and thrombotic events occurring during hospital stay. We also aimed to describe strategies used for thromboprophylaxis and to assess the characteristics of patients who received different doses heparin, either prophylactic or sub-therapeutic/therapeutic dosage.

## Methods

The START-COVID-19 Register starts in May 2020 after the widespread of the SARS2-COVID-19 pandemic in the frame of the START Register (NCT 02219984) [21]. This is a retrospective, observational, nationwide, multicentre register aimed to collect data on the clinical characteristics, laboratory findings, and drugs employed in patients infected by SARS2-COVID-19 virus, hospitalized in ordinary wards. Patients requiring ICU at admission were excluded from the study. The registry has been approved by the Ethical Committee of the Institution of the Coordinating Member (Azienda Ospedaliero-Universitaria, Policlinico S. Orsola-Malpighi, Bologna, Italy), and by all Ethical Committees of participating centers. Twenty-two hospitals distributed throughout Italy participate in these data collection.

The Registry is aimed to record local practice, therefore no specific tests or treatments were mandated by the study protocol. All patients underwent to nasopharyngeal and oropharyngeal swab on admission, and the presence of SARS2-COVID-19 infection was detected by polymerase chain reaction (PCR) method. The study focused in particular to the type and dosage of the thromboprophylaxis used, the occurrence of adverse thrombotic and bleeding events, and of death. A dedicated web-based case report form (e-CRF) obtained with “Electronic Data Capture” (EDC) system, based on the “Research Electronic Data Capture” online platform (REDCap, produced and distributed by Vanderbilt University and “REDCap Consortium”) [22]. The e-CRF collect demographic data, clinical data related to associated diseases, symptoms at admission, and type of treatment. The entity of associated comorbidity was measured using the Charlson’s comorbidity index [23]. Antithrombotic therapy was defined prophylactic when enoxaparin 4000–6000 U od, or fondaparinux 2.5 mg od, or nadroparin 2850-3800-5700 U od, or unfractioned heparin (UFH) 5000 U bid, were used. Treatment was defined sub-therapeutic/therapeutic when enoxaparin 4000 U bid, or enoxaparin 6000 U bid, or enoxaparin 8000 U bid, or fondaparinux 5–7.5 mg od, or nadroparin 3800 U bid or 5600 U bid, or UFH 12.500 U bid, were used. In addition, to evaluate the role of thromboprophylaxis in relation to age, we divided the patients taking into account the quartiles distribution of age. We decided to stratify patients into 2 classes: patients < 59 years (1° quartile) and patients with age ≥ 59 years (≥ 2° quartile).

Patients were followed-up during hospital stay, the follow-up ended when the patients was discharged, transferred to ICU, or died. The outcome was defined favourable when the patient was discharged, and severe when the patient was transferred to ICU or died. Thrombotic and bleeding events occurred during follow-up were recorded. Objective confirmation of thrombotic events was requested. Major bleeding (MB) was defined according with the International Society of Thrombosis and Haemostasis [24]. Clinically relevant non major bleedings (CRNMB) were defined as those events that are not major but require any kind of medical intervention [25].

### Statistical analysis

Descriptive analysis was performed. Continuous variables are expressed as median with interquartile range (IQR) or as mean plus or minus standard deviation (SD). Categorical variables are expressed as frequencies and percentages. Preliminary statistical analysis was performed using Wilcoxon signed-rank test (continuous variables) or Fisher exact test (categorical data). A *p* value < 0.05 was considered statistically significant.

A logistic univariate analysis was performed to estimate the association of heparin use and mortality. All significant variables were subsequently entered into a multivariable analysis. Risk was expressed as odds ratio (OR) with 95% CI. A 2-sided value of *p* < 0.05 was chosen for statistical significance.

We used the SPSS version 26 software (SPSS Inc, Chicago, IL, USA) and the Stata version 14 software (Stata Corp, College Station, TX) for Windows for data processing.

## Results

### Patients and thromboprophylaxis

**Fig. 1:**
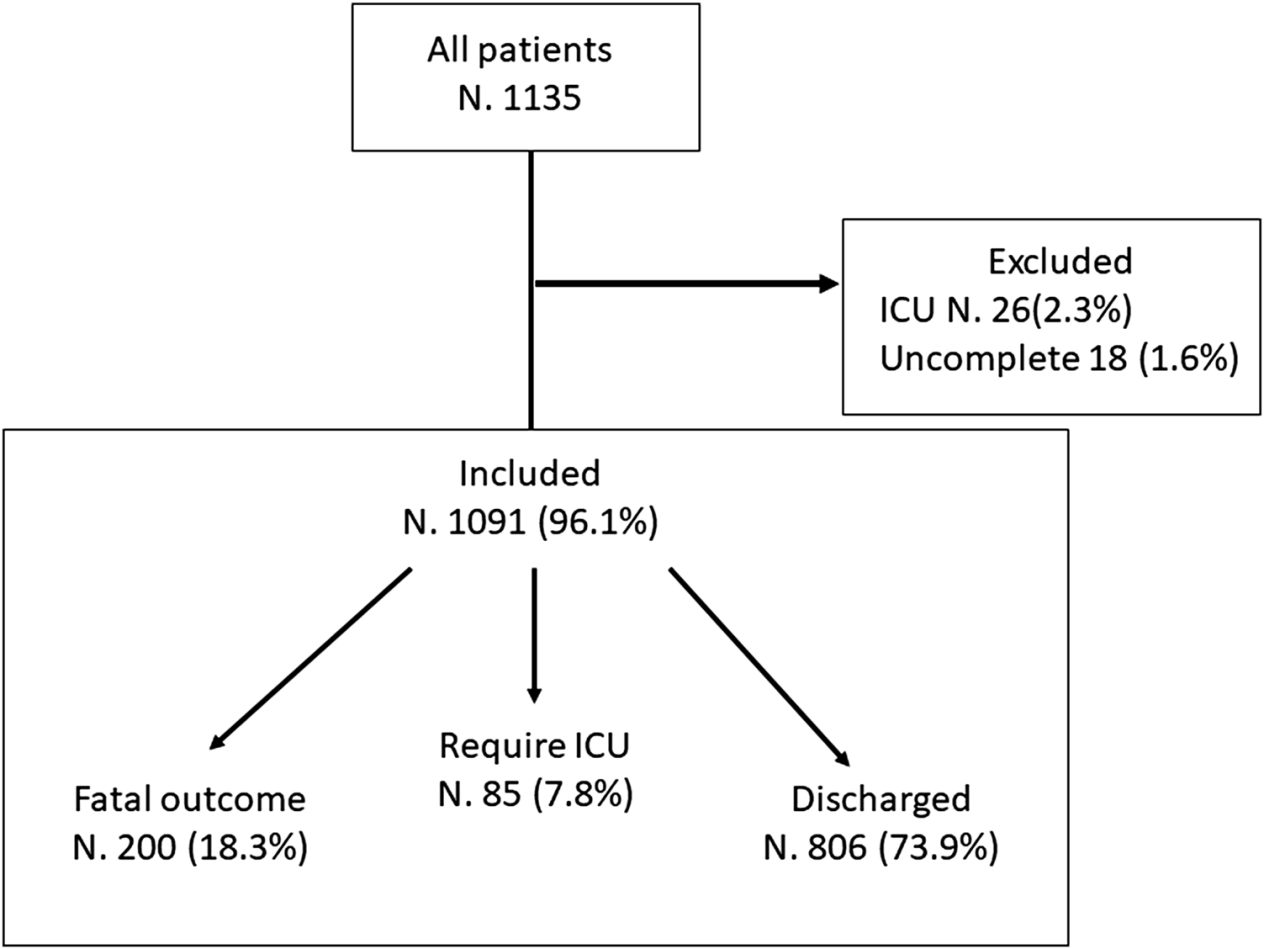
Flow-chart of the study

From March 1st and June 30th 2020, 1135 patients hospitalized for COVID-19 infection were included in the START-COVID-19 Register. The flow chart of the study is available Fig. 1; 1091 patients were included (59.9% males), with a median age of 71 years (IQR 59–82 years). Characteristics of the patients are detailed in Table 1. In particular, hypertension was present in 570 (52.2%) patients, the median Charlson’s index of the cohort was 3 (range 2–5), and 406 (37.2%) patients had no associated comorbidities. At admission, fever was present in 796 patients (73.0%), dyspnoea in 581 (53.3%), and cough in 450 (41.2%).

**Table 1:** Clinical characteristics of patients

	*N* (%)
Patients	1091
Males	653 (59.9)
Median age (IQR)	71 (59–82)
Median age males (IQR)	69 (58–80)
Median age females (IQR)	76 (63–85)
Body mass index	26 (24–29)
Hypertension	570 (52.2)
Atrial fibrillation	83 (7.6)
History of venous thromboembolism	36 (3.3)
Coronary artery disease	110 (10.1)
Heart failure	22 (2.0)
Peripheral obstructive arterial disease	16 (1.5)
Cerebrovascular disease	65 (6.0)
Neurological disease	145 (13.3)
Chronic obstructive pulmonary disease	112 (10.3)
Rheumatologic disease	21 (1.9)
Diabetes mellitus	190 (17.4)
Renal failure (eGFR < 30 mL/min)	261 (23.9)
Active Cancer	141 (12.9)
Median Charlson’s Index (IQR)	3 (2–5)
No comorbidities	406 (37.2)

During hospitalization, 508 (46.6%) patients received antiviral treatment, mainly lopinavir/ritonavir [379/508 patients (74.6%)]; 851 (78.0%) patients hydroxychloroquine, and 240 (22%) desametasone. At admission, 51 (4.7%) patients were on oral anticoagulants: 35 (68.6%) with vitamin K antagonists (VKAs) and 16 (31.4%) with direct oral anticoagulants (DOACs) (Table 2); 23 patients were already receiving low-molecular weight heparin (LMWH) at prophylactic dosage.

**Table 2:** Antithrombotic and anticoagulant treatment during hospital stay

	*N* (%)
*N*. Patients	769 (70.7)
LMWH/fondaparinux	754 (98.0)
Prophylactic dosage^a^	607 (78.9)
Therapeutic-subtherapeutic dosage^b^	147 (19.1)
Oral anticoagulants	15 (2.0)
Type of drug	
Enoxaparin	706 (92.3)
Nadroparin	16 (2.1)
Fondaparinux	19 (2.5)
UFH sc	9 (1.2)
Oral anticoagulants	15 (2.0)

^a^Enoxaparin 4000–6000 U od; fondaparinux 2.5 mg od; nadroparin 2850–3800-5700 U od, unfractioned heparin (UFH) 5000 U bid

^b^Enoxaparin 4000 U bid, enoxaparin 6000 U bid, enoxaparin 8000 U bid; fondaparinux 5 mg or 7.5 mg od; nadroparin 3800 U bid, 5600 U bid; UFH 12.500 U bid

During hospital stay, 769 (70.7%) patients were treated with antithrombotic drugs: 15 (2.0%) patients continued treatment with oral anticoagulants, and 754 (98.0%) were treated with LMWH/fondaparinux/unfractioned heparin (UFH). Among these patients, 607 (78.9%) received prophylactic dosage, 93 (12.1%) received sub-therapeutic dosage, and 69 (9.0%) therapeutic dosage. The great majority of patients were treated with enoxaparin (92.3%). The characteristics of patients treated with prophylactic dosage and with sub-therapeutic/therapeutic dosage are reported in Table 3. Atrial Fibrillation (AF), previous thromboembolism, neurological disease, and cancer were less frequent in patients who did not receive prophylaxis in comparison to patients who received prophylaxis. Accordingly, in the latter group the Charlson’s score was significantly higher. Patients who received antithrombotic treatment at sub-therapeutic/therapeutic dosage, had higher prevalence of AF and coronary artery disease with respect to patients who received prophylactic dosage (Table 4).

**Table 3:** Characteristics of patients with and without thromboprophylaxis

	Prophylaxis	NO Prophylaxis	*p* value
Patients	769 (70.5)	322 (29.5)	
Males	457 (59.4)	196 (60.9)	0.7
Median age (IQR)	72 (60–83)	70 (56–81)	0.3
Hypertension	414 (46.2)	166 (51.6)	0.1
Atrial fibrillation	74 (9.6)	9 (2.8)	0.000
History of venous thromboembolism	32 (4.2)	4 (1.2)	0.01
Coronary artery disease	86 (11.2)	24 (7.5)	0.08
Heart failure	19 (2.5)	3 (0.9)	0.1
Peripheral obstructive arterial disease	15 (2.0)	1 (0.3)	0.05
Cerebrovascular disease	50 (6.5)	15 (4.7)	0.3
Neurological disease	126 (16.4)	19 (5.9)	0.000
Chronic obstructive pulmonary disease	96 (12.5)	16 (5.0)	0.000
Rheumatological disease	19 (2.5)	2 (0.6)	0.05
Liver disease	15 (2.0)	4 (1.2)	0.6
Diabetes mellitus	143 (18.6)	47 (14.6)	0.1
Renal failure (eGFR < 30 mL/min)	67 (8.7)	29 (9.0)	0.8
Cancer	111 (14.4)	30 (9.3)	0.02
Median Charlson’s Index (IQR)	4 (2–5)	3 (1–4)	0.007
No comorbidities	276 (35.9)	130 (40.4)	0.2
Major bleeding	9 (1.2)	–	
CRNMB	9 (1.2)	–	
Total bleeding	18 (2.4)	–	
Death	127 (16.5)	73 (22.7)	0.02

**Table 4:** Clinical characteristics and outcomes of patients according to the intensity of antithrombotic treatment

	LMWH (*) prophylactic dosage	LWMH/OA (**) Therapeutic/sub-therapeutic dosage	
Patients	607 (78.9)	162 (21.1)	
Males	364 (60.0)	93 (57.4)	0.6
Median age (IQR)	72 (60–82)	64 (62–86)	0.2
Hypertension	275 (45.3)	82 (50.6)	0.4
Atrial fibrillation	47 (7.7)	27 (16.7)	0.001
Venous thromboembolism	19 (3.1)	13 (8.0)	0.01
Coronary artery disease	59 (9.7)	27 (16.7)	0.02
Heart failure	13 (2.1)	6 (3.7)	0.3
Peripheral obstructive arterial disease	39 (6.4)	11 (6.8)	0.3
Liver disease	13 (2.1)	2 (1.2)	0.7
Neurological disease	92 (15.2)	34 (21.0)	0.07
Chronic obstructive pulmonary disease	72 (11.9)	24 (14.8)	0.4
Diabetes mellitus	104 (17.1)	39 (24.1)	0.06
Renal failure (eGFR < 30 mL/min)	46 (7.6)	13 (8.0)	0.9
Cancer	82 (13.5)	29 (17.9)	0.2
Rheumatological disease	92 (15.2)	34 (21.0)	0.6
Median Charlson’s Index (IQR)	3 (2–5)	4 (2–5.75)	0.000
No co-morbidities	234 (38.6)	42 (25.9)	0.003
Major bleeding	4 (0.7)	5 (3.1)	0.02
CRNMB	3 (0.5)	6 (3.7)	0.003
Total bleeding	7 (1.1)	11 (6.8)	0.000
Death	94 (15.5)	33 (20.4)	0.2

(*)Enoxaparin 4000–6000 U od; fondaparinux 2.5 mg od; nadroparin 2850–3800-5700 U od, unfractioned heparin (UFH) 5000 U bid

(**)Enoxaparin 4000 U bid, enoxaparin 6000 U bid, enoxaparin 8000 U bid; fondaparinux 5 mg or 7.5 mg od; nadroparin 3800 U bid, 5600 U bid; UFH 12.500 U bid

### Bleeding and thrombotic complications

During hospital stay, 9 (1.2%) patients had a major bleeding (MB) and 9 (1.2%) patients a clinically relevant non major bleeding (CRNMB). All bleedings occurred among patients treated with antithrombotic drugs. MBs and CRNMBs occurred more frequently among patients treated with sub-therapeutic/therapeutic dosage with respect to patients treated with prophylactic dosage [5 (3.1%) and 6 (3.7%) vs 4 (0.7%) and 3 (0.5%), respectively] (Table 4). Fifty-nine patients had venous thromboembolism during observation (5.4%), 18 (30.5%) patients had deep vein thrombosis (DVT), 39 (66.1%) patients had pulmonary embolism (PE), and 2 (3.4%) patients had DVT + PE.

### Clinical deterioration and mortality

Favourable outcome was reported in 806/1091patients (73.9%). Among patients with severe outcome, 85 (7.8%) were transferred to ICU, due to deterioration of respiratory failure, and 200 patients (18.3%) died (Fig. 1). Clinical characteristics of patients with fatal outcome and of discharged patients are reported in Table 5. Patients with fatal outcome were significantly older with respect to patients discharged, showing higher prevalence of hypertension, of coronary artery disease, of peripheral artery obstructive disease (POAD), of cerebrovascular and neurological disease, of cancer, and a higher median Charlson’s comorbidity index (Table 5). Patients who did not had comorbidities at admission were significantly less represented among patients who died with respect to patients with favourable outcome. Laboratory tests showed that median D-dimer levels and PT ratio were significantly higher in patients with fatal outcome with respect to discharged patients (data not shown).

**Table 5:** Clinical characteristics of patients with fatal outcome

	Dead patients *N* (%)	Alive *N* (%)	*p* value
Patients	200 (18.3)	891 (81.7)	
Males	124 (62.0)	529 (59.4)	0.5
Median age (IQR)	83 (76–88)	68 (57–69)	0.000
Body mass index (IQR)	26 (24–29)	26 (24–29)	0.9
Hypertension	123 (61.5)	447 (50.2)	0.004
Atrial fibrillation	21 (10.5)	62 (7.0)	0.1
Venous thromboembolism	8 (4.0)	28 (3.1)	0.5
Coronary artery disease	34 (17.0)	76 (8.5)	0.001
Heart failure	7 (3.5)	15 (1.7)	0.1
Peripheral obstructive arterial disease	9 (4.5)	7 (0.8)	0.001
Cerebrovascular disease	20 (10.0)	45 (5.1)	0.01
Neurological disease	54 (27.0)	91 (10.2)	0.000
Chronic obstructive pulmonary disease	31 (15.5)	81 (9.1)	0.01
Rheumatologic disease	3 (1.5)	18 (2.0)	0.8
Liver disease	4 (2.0)	15 (1.7)	0.8
Diabetes mellitus	46 (23.0)	144 (16.2)	0.02
Cancer	44 (22.0)	97 (10.9)	0.000
Renal failure (eGFR < 30 mL/min)	43 (36.1)	53 (9.9)	0.000
Charlson’s Index (IQR)	5 (4–6)	3 (2–4)	0.000
No comorbidities	53 (26.5)	353 (39.6)	0.000

To evaluate the association of fatal outcomes with antithrombotic treatment, we performed a logistic regression multivariable analysis on patients considering the different quartiles of age. Antithrombotic treatment resulted independently associated with survival in patients aged ≥ 59 years (≥ 2° quartile) (Table 6).

**Table 6:** Multivariate analysis for death in patients aged ≥ 59 years

	OR (95% CI)
Age	1.1 (1.1–1.1)
Thromboprophylactic treatment (all dosages)	0.4 (0.3–0.6)
Peripheral Obstructive Arterial Disease	4.9 (1.6–15.1)
Neurological disease	1.6 (1.0–2.5)

## Discussion

In this study, we describe the characteristics of 1091 patients admitted to general wards for laboratory confirmed infection by COVID-19 during the first wave of the pandemic in Italy, focusing the attention on the antithrombotic treatment and its association with patient outcomes. Mortality of hospitalized patients with COVID-19 varies significantly among the published case series, ranging from 16% to more than 50% [6, 26–28]. In our cohort the mortality rate was 18.3%, higher than that reported from other studies conducted in China [29, 30]. However, it was similar to the case fatality rate observed by Wu C et al. [31] in the early period of the epidemic in China, in New York [8] and in Italy [1, 7, 32], In particular the CORIST study [32], conducted in Italian hospitals, reported almost the same mortality rate as in our study. The variability of the mortality rate described in the studies can be explained by different organization of hospitals and of out-of-hospital health services, leading to a different severity of patients for whom hospital admission is required. In any case, the mortality rate is very high when patients require treatment in ICU wards.

Overall, published data identify advanced age as the principal risk factor for mortality, and for this reason the ongoing vaccination campaigns are directed firstly to the elderly subjects. In our cohort, patients with fatal outcome had a median age of 83 years, confirming the previous finding, instead a median age of 68 years was reported for survivors.

In our patients, several other risk factors have been associated with fatal outcome, in particular hypertension, coronary artery disease, diabetes mellitus, active cancer, chronic obstructive pulmonary disease, and renal failure, as described from other authors [10].

In our cohort 769 (70.7%) patients were treated with antithrombotic drugs during hospital stay, mainly with enoxaparin, that was used in 92.3% of patients. These patients were more frequently affected by comorbidities, such as hypertension, AF, previous thromboembolism, neurological disease, peripheral artery obstructive disease (POAD), and cancer with respect to patients who did not receive thromboprophylaxis. Despite the elevated number of associated risk factors, the mortality rate in this group was lower than that recorded in patients who did not receive heparin. This finding was confirmed at multivariate analysis especially when older patients, aged ≥ 59 years, were considered. The protective effect of heparin was not detected in patients < 59 years, who showed a low mortality rate (only 4 patients had fatal outcome in this group). The presence of POAD and of neurological diseases were also independently associated with mortality, probably as a consequence of the high frequency of coronary artery disease and renal failure usually associated with these clinical conditions.

Patients treated with heparin at prophylactic dosage showed a lower bleeding risk, in comparison to patients treated with sub therapeutic/ therapeutic dosages. The increased bleeding risk associated with the use of sub therapeutic/ therapeutic dosages of heparin has been reported by other authors [19, 20], and is one of the reasons why prophylactic intensity anticoagulation remains the recommended strategy, waiting for the results of ongoing randomized controlled trials [16, 18], even if a higher-intensity anticoagulation can be considered for patients judged to be at high thrombotic and low bleeding risk.

Due to the severe pandemic diffusion, with a large number of patients requiring hospitalization, VTE events were diagnosed only when clinically evident. No systematic tests were performed to exclude asymptomatic DVT. Moreover, we cannot exclude that PE could be the cause of fatal outcome in patients with severe respiratory failure, because no systematic autoptic examination was performed.

We acknowledge the limitations of our study. Firstly, it is a retrospective observational study, and the treatment used are in line with the local practice, without any indication by the study protocol. The study was designed to evaluate the type and dosage of heparin used, therefore the availability of data is high. However, the wide range of dosages used, imposes to analyze patients in two groups that include different dosages. Moreover, the severity of patients enrolled may be influenced by the availability of ICU beds that could have limited the number of patients who received mechanical ventilation. Strength of the study are the multicentric design, and the accuracy and completeness of follow-up for all patients enrolled.

In conclusion, our data confirmed the high mortality rate of hospitalized patients affected by COVID-19 infection, even when the presentation of the disease was moderately severe leading to admission to general wards. Risk factors for fatal outcome were older age, associated POAD and neurological disease. Treatment with antithrombotic drugs was significantly associated with a 60% reduction of mortality rates. Patients who received sub therapeutic/ therapeutic dosages showed a significantly higher bleeding risk.
